# The Role of Nonsteroidal Anti-Inflammatory Drugs (NSAIDs) in the Management of the Post-Embolization Symptoms after Uterine Artery Embolization

**DOI:** 10.3390/ph3061729

**Published:** 2010-05-26

**Authors:** Tiago Bilhim, João Martins Pisco

**Affiliations:** 1 Saint Louis Hospital, Interventional Radiology / Rua Luz Soriano, 182, Lisbon 1200-249, Portugal; E-Mail: joao.pisco@chln.min-saude.pt (J.M.P.); 2 Departamento de Anatomia, Faculdade de Ciências Médicas (FCM), Universidade Nova de Lisboa, 1169-056, Lisboa, Portugal; 3 Departamento de Radiologia, Faculdade de Ciências Médicas (FCM), Universidade Nova de Lisboa, 1169-056, Lisboa, Portugal

**Keywords:** uterine artery embolization, post-embolization syndrome, nonsteroidal anti-inflammatory drugs

## Abstract

Uterine artery embolization (UAE) is usually a very painful procedure. Although pain after the procedure can occur as a single symptom, it usually is associated with other symptoms such as nausea, vomiting, pelvic pain, general malaise, fever and leukocytosis that characterize the post-embolization syndrome. Management of the post-embolization symptoms and of pain in particular, is paramount if UAE is to be performed as an outpatient procedure. Different protocols have used analgesic and/or anti-inflammatory agents to control these symptoms. Nonsteroidal anti-inflammatory drugs (NSAIDs) are frequently used in association with analgesic drugs to control post-embolization symptoms. In our institution the patients start oral medication with NSAIDs the day before the procedure and continue it during and after UAE. We also mix NSAIDs with the embolizing particles. This enables a reduction in the inflammation present in the uterine fibroids and helps controlling the pain. The purpose of this paper is to review the importance of NSAIDs in the management of the post-embolization symptoms. We describe the protocol that we use in our institution that enables us to perform the procedure on an outpatient basis with same day discharge and good control of the post-embolization symptoms with oral NSAIDs and analgesics.

## 1. Introduction

Uterine artery embolization (UAE) is usually a painful procedure. In the first hours after the procedure many patients suffer from post-embolization syndrome, consisting of nausea, vomiting, pelvic pain, low grade fever, fatigue, general malaise and leukocytosis [1]. The pain is due to fibroid ischemia and transient ischemia of the normal myometrium. It is expected that there will be some correlation with the volume of tissue infarcted and the severity of this syndrome. However, the severity of pain is largely unpredictable [2]. 

Management of these post-embolization symptoms and of the associated pain in particular, is paramount if UAE is to be performed as an outpatient procedure [3]. Several different strategies, involving oral, intravenous, epidural, and/or patient-controlled analgesia, have been utilized in an attempt to manage the pain associated with UAE [3,4,5,6]. We are currently using a combination of nonsteroidal anti-inflammatory drugs (NSAIDs) before, during and after the procedure in order to decrease the pain after UAE, thus allowing the procedure to be performed on an outpatient basis. As opposed to the majority of the other institutions that perform UAE using intravenous narcotics for pain management we base our medication protocol on the anti-inflammatory and analgesic properties of NSAIDs.

## 2. Different Medication Protocols to Treat Pain after UAE

Besides pain and the other post-embolization symptoms associated with UAE in the first hours after the procedure, other symptoms such as abdominal swelling, constipation, fever, vaginal bleeding, pain, nausea/vomiting, anorexia or fatigue can occur on the following days. As most patients will experience 4 to 5 days of post-procedural symptoms, most centres where UAE is performed admit their patients in the hospital for 1 to 2 days after the procedure for abdominal pain or vomiting control [5]. Since the first reports on UAE for the treatment of uterine fibroids, most subsequent studies have also indicated that patients should be hospitalized at least overnight for pain control. However, based on efficient pain-control medication protocols some authors have started to perform UAE sending the patients home on the day of the procedure [4].

Many different agents such as fentanyl, ibuprofen, promethazine, acetaminophen, codeine, meperidine, ketorolac, cefazolin, hydroxyzine, oxycodone, morphine, droperidol, ondasetron, midazolam and hydrocodone have been used in more or less complex associations with variable results [3,4,5,6]. Baerlocher *et al.* [3] used morphine sulphate administered through a patient-controlled analgesic (PCA) pump. After discharge pain was controlled with NSAIDs and narcotic analgesic drugs. Klein and Schwartz [4] used ketorolac, cefazolin, meperidine, hydroxyzine, ibuprofen, promethazine, oxycodone and acetaminophen given orally, and morphine i.v. for severe non-responding pain. Droperidol or ondasetron were the anti-emetic drugs used. Ryan *et al.* [6] used fentanyl i.v. in continuous infusion before, during and after the procedure, and oral ibuprofen and intravenous promethazine after UAE. The morning after UAE they used oral analgesia with oxycodone hydrochloride. Siskin *et al.* [5], developed a protocol in combination with the pain service and anaesthesiology departments at their institution, using ketorolac i.v. after each uterine artery embolization, with additional fentanyl i.v., meperidine i.v., hydroxyzine i.v. and ketorolac i.v.. Hydrocodone plus acetaminophen and ibuprofen were given by mouth. Postprocedural pain was assessed using the Graphic Rating Scale, assigning a number from 0 to 10 to correspond to the sensation of no pain (0) or the worst pain ever experienced (10). They had a mean immediate postprocedural pain score of 5.7 (range, 1–9). Forty-seven out of 49 (96%) patients were treated as outpatients with good pain control 6 to 8 hours after UAE. Rasuli *et al.* [7] used superior hypogastric nerve block (SHNB) in addition to morphine tablets and indomethacin or naproxen rectal suppositories and proved that this protocol achieved good pain control, enabling the procedure to be performed on an outpatient basis with minimum pain. Before discharge, each patient’s pain level was recorded on a simple four-point descriptive scale (no pain, mild, moderate, and severe pain) by a nurse. All patients could be discharged within 6 hours after the procedure (median time, 5 hours; range, 4.5–6 hours) and, at the time of discharge, reported mild pain or no pain. Lampmann *et al.* [8] reinforced the idea that proper pain management starts prior to the procedure, and not after the first pain is experienced by the patient during or just after the procedure itself. One hour prior to the procedure kefsol i.v. and dipidolor i.v. on a PCA pump were used. After UAE the authors used the PCA pump with dipidolor plus diclophenac and paracetamol as suppositories. This protocol was performed as an inpatient procedure. 

Another agent that has been previously used is ketoprofen [9]. Ketoprofen i.m. has been used one hour before UAE and on the following days after the procedure with good results in the control the post-embolization pain.

Different strategies have used analgesic and/or anti-inflammatory agents along with the embolizing agents. Pain control has also been addressed using ibuprofen-loaded microspheres for uterine artery embolization [10,11]; mixing the embolizing agent with ketorolac [12] or lidocaine [9]; or injecting lidocaine directly intra-arterially (although this practice has been abandoned due to the induced arterial vasospasm). 

Despite the different combinations of medications used, most institutions rely on morphine/morphine-derived analgesics through a PCA pump to control post-UAE pain and admit their patients overnight in order to better control the symptoms. NSAIDs are usually used as a second hand drug to treat pain after intravenous narcotics. 

## 3. Medication Protocol to Treat UAE Post-Embolization Symptoms in Our Institution

We have shown that using prophylactic pre-embolization medication with NSAIDs for pain, allows UAE to be performed safely as an outpatient procedure with high patient satisfaction rates, good post-embolization symptom control, without risking the medium-term results [13] (Copyright Elsevier, Society of Interventional Radiology, 2009).

The patients start oral medication at home on the day prior to UAE. They are medicated twice a day (at breakfast and dinner) with an acid suppressing drug (omeprazole 20 mg—Bluepharma), an anti-inflammatory (naproxen 1000 mg, Naprosyn, Roche), an anti-histaminic (hydroxyzine 25 mg, Atarax, UCB) and a stool softener (sodic docusate and sorbitol 10 mg + 1340 mg as suppositories, Clyss—Go, Prospa). They repeat the medication at home on the day of UAE (at breakfast).

Patients are usually admitted to the hospital on the morning of embolization. They are first directed to the hospital ward were a receiving nurse checks vital signs and applies an i.v. access. Before the procedure a saline drip infusion is started. Still in the ward room, just before embolization the patients have an anxiolytic (diazepam 5 mg sub lingual, Ratiopharm), an acid suppressing drug (omeprazole 20 mg i.v), analgesics (metamizol 2 g i.v, Nolotil, Boehringer Ingelheim and tramadol 100 mg i.v, Tramal, Grunental) antiemetics (metoclopramide 25 mg i.v, Primperan, Sanofi—Aventis and Droperidol 0.10 mg i.v., Janssen Cilag) an anti-inflammatory (piroxicam 20 mg i.v., Feldene, Pfizer), and an antibiotic (cefazoline 1 g i.v., Bristol Myers Squibb).

The patients are then directed to the angiography suite and the procedure is started. Both uterine arteries are embolized with nonspherical polyvinyl alcohol (PVA) particles mixed with ketoprofen 100 mg. During embolization an anti-inflammatory (ketorolac trometamine 30 mg i.v. Toradol, Roche) is administered twice before the embolization of each uterine artery (total amount of 60 mg). Midazolam 1 mg i.v. is given if necessary for anxiety control. 

When the procedure is finished, the patients are redirected to the ward room where a dedicated nurse checks for vital signs and post-embolization symptoms. Medication is continued using an acid suppressing drug (omeprazole 20 mg i.v.) analgesics (paracetamol 1g i.v. and metamizol 2 g i.v.), anti-inflammatory (ketorolac tromethamine 30 mg i.v. and piroxicam 20 mg i.v.) and antiemetics (metoclopramide 25 mg i.v. and ondasetron 2 mg i.v., Hikma). 

Two to three hours after the procedure, all asymptomatic patients and those with mild symptoms, without nausea or vomiting have a light meal, start oral medication with tramadol 37.5 mg plus paracetamol 325 mg (Zaldiar, Grunenthal) and are discharged from the hospital, 4 to 8 hours after the procedure. Just before discharge, tramadol 100 mg i.v. plus metoclopramide 25 mg i.v. are given to every patient. 

Four to eight hours post-embolization patients with no symptoms, mild to severe pain, only light fatigue, anorexia, abdominal swelling or minor vaginal bleeding are considered for discharge.

The night of embolization, at home, the patients have omeprazole 20 mg, hydroxyzine 25 mg and naproxen 500 mg. As on-demand medication, the patients are given tramadol 37.5 mg plus paracetamol 325 mg (Zaldiar) and codeine 30 mg plus paracetamol 500 mg (Dol-U-Ron, Neo-Framaceutica) by mouth for pain and dimenhydrinate 100 mg (Enjomin, Codilab) as suppositories for vomiting. The day after the procedure the patients start oral medication with omeprazole 20 mg twice a day, naproxen 500 mg twice a day and Clyss Go (as suppositories) and are advised not to stay in bed. 

We measure pain using a numeric pain score scale rated from 0 (sensation of no pain) to 10 (the worst pain). The patients are asked to rate verbally their pain severity. During embolization there is a mean pain score of 0.9, after embolization and before discharge the mean pain score is 2.5. At discharge the mean pain score is 0.9. At home, on the night of discharge, the mean pain score is 1.1. On the morning following embolization the mean pain score is 0.7. 

**Figure 1:**
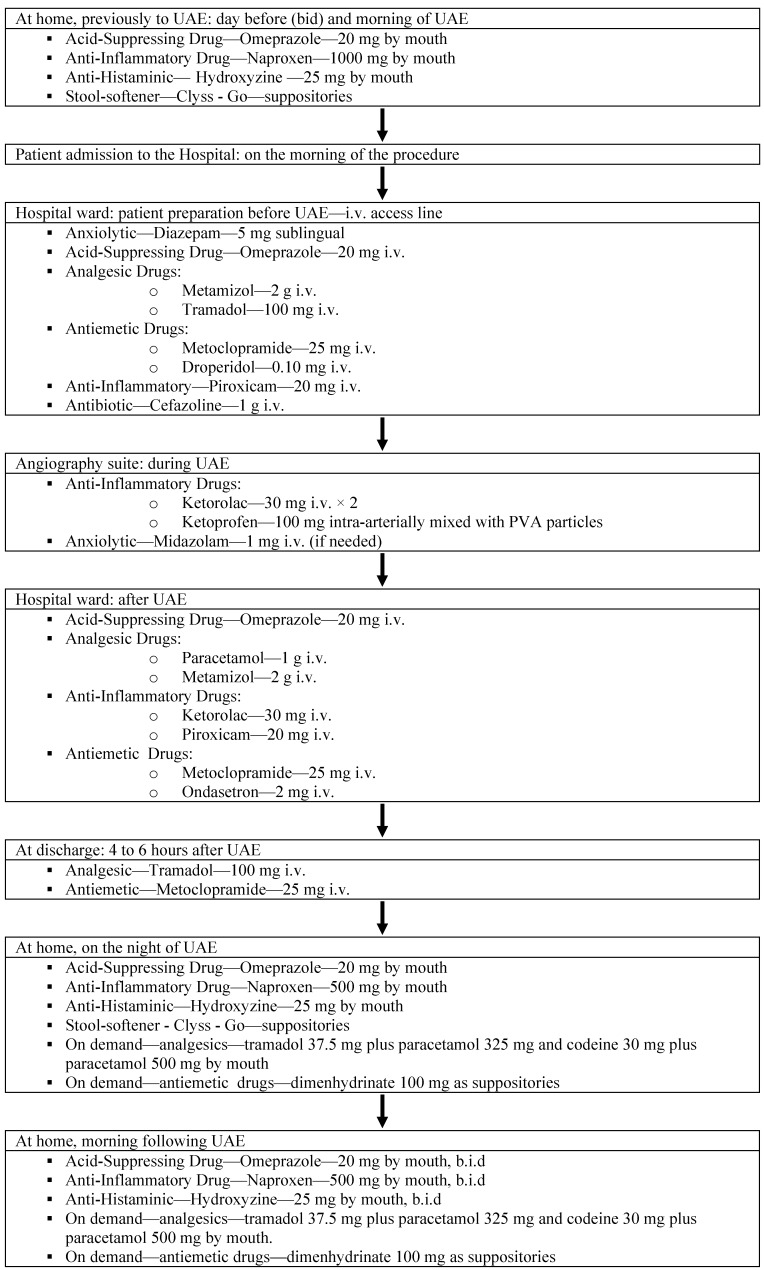
UFE pain management algorithm.

We have treated over 900 patients using this protocol and currently perform the procedure on an outpatient basis in the vast majority of cases. Only patients with persisting pain and/or vomiting 6–8 hours after UAE are admitted overnight. The use of oral NSAIDs is cornerstone in the pain management before and after UAE allowing us to treat patients without relying on morphine PCA pumps. Figure 1 summarizes the protocol used.

## 4. Role of Nonsteroidal Anti-Inflammatory Drugs (NSAIDs) in the Management of UAE Post-Embolization Symptoms

As shown, we rely on NSAIDs before, during and after UAE to control symptoms. We only rely on analgesic drugs in the immediate time before and after UAE. Afterwards, the NSAIDs play a major role in symptom control. We only use analgesic drugs as on-demand medication when symptoms persist. 

NSAIDs include a vast variety of different agents with three main effects—anti-inflammatory, analgesic and antipyretic. Most NSAIDs act as nonselective inhibitors of the enzyme cyclooxygenase (COX), inhibiting both the cyclooxygenase-1 (COX-1) and cyclooxygenase-2 (COX-2), which decreases production of pro-inflammatory prostaglandin precursors. In our pain management protocol we use four different NSAIDs—naproxen, piroxicam, ketorolac and ketoprofen.

Naproxen is a NSAID that works by inhibiting both the COX-1 and COX-2 enzymes, with a bioavailability of 95% (oral), 99% protein binding, with hepatic metabolism, renal excretion and a half life of 12–24 hours. It is commonly used for the reduction of pain, fever, inflammation and stiffness. We use oral naproxen 1,000 mg on the day before and on the morning of UAE and continue it on the night of UAE and on the following days (500 mg). 

Piroxicam is a non-selective COX inhibitor possessing both analgesic and antipyretic properties. It undergoes enterohepatic circulation, with 4–10% renal metabolism and excretion, with a half life of 30–86 hours. It is used to relieve the symptoms of rheumatoid and osteoarthritis, primary dysmenorrhoea, and postoperative pain, acting as an analgesic, especially where there is an inflammatory component. Piroxicam 20 mg is administered i.v. immediately before and after UAE, based on the good analgesic effects when managing inflammation-associated pain as in post-UAE. 

Ketorolac or ketorolac tromethamine is a non-selective COX inhibitor in the family of heterocyclic acetic acid derivatives, often used as an analgesic, antipyretic, and anti-inflammatory, with a bioavailability of 100% by all routes, with hepatic metabolism, renal and biliary excretion and a half life of 3.3–9.2 hours. We use ketorolac trometamine i.v. during and immediately after UAE based on its rapid acting time, excellent bioavailability and short half life, enabling good pain control, quickly and for short periods of time. 

Ketoprofen is a propionic acid-derived non steroidal anti-inflammatory drug employed as an analgesic and anti-inflammatory agent that can be given by mouth, topically, i.v. or i.m. with anti-inflammatory, analgesic and antipyretic effects. Ketoprofen is a non-selective COX inhibitor, inhibiting COX-1 and COX-2 enzymes reversibly, with 99% protein binding and a half life of 2–2.5 hours. Based on its rapid acting time and short half-life we use it for intra-procedural pain control mixed with the embolizing agent (PVA) in the embolizing solution cup.

We also use paracetamol, that could be considered a NSAID, but paracetamol has very little anti-inflammatory effect in many tissues. We use it based on its analgesic properties and not on the anti-inflammatory effects. It is commonly used for the relief of headaches, and other minor aches and pains. In combination with opioid analgesics, paracetamol can be used also in the management of more severe pain as in post-UAE. 

## 5. Discussion

In medical procedures there is a trend to shift toward ambulatory care, both to increase patient satisfaction and to reduce health care costs. To perform UAE as an outpatient procedure it is important to control the post-embolization symptoms, particularity pain and vomiting and the patients should be informed about those symptoms and the corresponding medication, thus, an effective post-embolization medication must be provided [3]. 

We agree with Lampmann *et al.* [8] in that proper pain management must be addressed before the procedure, and not only after the first pain is experienced by the patient. We think that that the use of NSAIDs before the procedure (naproxen and piroxicam) is essential to control pain during and after UAE, reducing the inflammation present in the fibroid. During UAE we rely on the anti-inflammatory drugs ketorolac and ketoprofen, and afterwards we use ketorolac and piroxicam. The patients remain on NSAID therapy with naproxen in the days following UAE to reduce the inflammation/ischemia related pain. 

During and just after UAE one of the chosen NSAIDs is ketorolac. As suggested by Siskin *et al.* [5], ketorolac has potent analgesic and moderate anti-inflammatory activity and may potentiate the action of the tramadol given previously. Due to the administration of ketorolac just before the embolization of each uterine artery, few of our patients report any pain during the procedure. Siskin *et al.* [5] administered ketorolac after the embolization of each uterine artery but we do it beforehand and we continue it after the embolization. After performing the embolization, metamizol, paracetamol, piroxicam and ketorolac are administered sequentially. 

Another agent used during UAE is ketoprofen. We found that combining the strong anti-inflammatory activity of ketoprofen and the ability to mix it with the PVA particles in the embolizing solution could be used to relieve pain after UAE. We described this new form of use of a NSAID that allows good control of pain during and after UAE [14] (Copyright Elsevier, Society of Interventional Radiology, 2009). Our results showed a reduction in post-embolization pain during the first eight hours following the procedure in those patients treated with ketoprofen mixed with the PVA particles. This can be easily explained by the short half life of ketoprofen (2–2.5 hours), making this an ideal agent to be used during UAE that acts quickly allowing efficient pain control during short periods of time. The mean post-embolization pain for patients treated with PVA mixed with ketoprofen was less severe than the one for patients treated with PVA alone. Thus, PVA particles mixed with ketoprofen helps relieving the pain during the first eight hours post-embolization, with no effect in the post-embolization pain after discharge, or on the clinical outcomes at 6 months. 

As apposed to the short-time-acting ketoprofen used at the time of UAE, we use naproxen 1000 mg by mouth and start it on the day before UAE because it has a half life of 12 to 15 hours so that when patients start UAE are already under the anti-inflammatory action. This protocol allows sufficient time for the anti-inflammatory properties of the oral naproxen used to take action. During and after embolization and before discharge most patients do not feel any pain proving the efficacy of the anti-inflammatory medication started on the day before and the analgesic association given during and after the procedure. Our mean pain score in the first 4–6 hours post UAE is 2.5 (scale 0–10), which is significantly lower than the 5.7 mean pain score reported by Siskin *et al.* [5], proving the efficacy of our pain-management protocol.

Patients start anti-inflammatory drugs before embolization to decrease the inflammation that is present in almost all fibroids and thus the post-embolization symptoms. Besides that, the anti-inflammatory drugs have some analgesic action. We believe that continuing the anti-inflammatory medication with naproxen after discharge is paramount in order to avoid late development of pain in the night after UAE. As we have shown mean pain score at discharge is usually low (0.9), but at home, on the night of discharge, the mean pain score increases slightly (1.1). Discontinuation of medication has leaded some patients to late night hospital readmissions due to intense pain. When patients are compliant with the medication, there is usually good pain control at the hospital during and after UAE and at home after UAE. 

In order to reduce vomiting the patients start an acid-suppressing drug (omeprazole) that is a proton pump inhibitor before embolization. Vomiting is due to embolization but also to the effect of analgesic and anti-inflammatory drugs in gastric mucosa even if they are administered i.v. We use the acid suppressing medication on the day before UAE to decrease the effects of the anti-inflammatory on gastric mucosa and the vomiting that may be associated with the anti-inflammatory when administered by mouth or i.v. For further reduction of vomiting, omeprazole is associated with metoclopramide and droperidol (injected i.v.) before embolization and with metoclopramide and ondasetron (injected i.v.) after finishing UAE. After discharge the vomiting is controlled by dimenhydrinate (suppositories) as on-demand medication. 

Further studies are required in order to achieve optimal post-embolization pain control. In spite of good overall pain control achieved with this protocol, there is still great variability of pain experienced between different patients. The many NSAIDs and analgesics used with strong dosages require active gastric protection to reduce vomiting and may have some additional side affects. More so, the relatively complicated and long list of different drugs used make it difficult to achieve correct and timely administration in all patients and nurse staff must be at all times available to assist the patients after UAE. Forgetting even just one of the medications may have disastrous implications on the post-embolization symptoms, and patient compliance is paramount. We have used alternative pain-management protocols based on acupuncture [15], achieving similar pain control with only half the dose of NSAIDs and analgesics administered. There are no studies using only NSAIDs in the management of pain after UAE or comparing NSAIDs and analgesics. We think that new protocols based on less medication are needed allowing efficient pain control in all patients in a homogeneous fashion.

## 6. Conclusions

Most centres perform UAE with overnight hospital admission using morphine/morphine-derived analgesics through a patient-controlled analgesic pump in order to control post-UAE pain. We rely on NSAIDs before, during and after UAE to control symptoms, combining different types of NSAIDs and administering them in different ways (by mouth, i.v. and intra-arterially mixed with the embolizing agents). In this way we are able to deliver the anti-inflammatory and analgesic effects of NSAIDs before, during and after UAE and in both a systemic and localized fashion. This new form of treatment enables UAE to be performed on an outpatient setting with less pain and fewer post-embolization symptoms.
